# Supplementary material for: Improved split fluorescent proteins for endogenous protein labeling
Source: Nat Commun. 2017 Aug 29;8:370. doi: 10.1038/s41467-017-00494-8 (PMC5575300; doi:10.1038/s41467-017-00494-8)
Supplement: Supplementary file 1 — Supplementary Information [file 41467_2017_494_MOESM1_ESM.pdf]

Description of Supplementary Files

File Name: Supplementary Information
Description: Supplementary Figures and Supplementary Tables

a

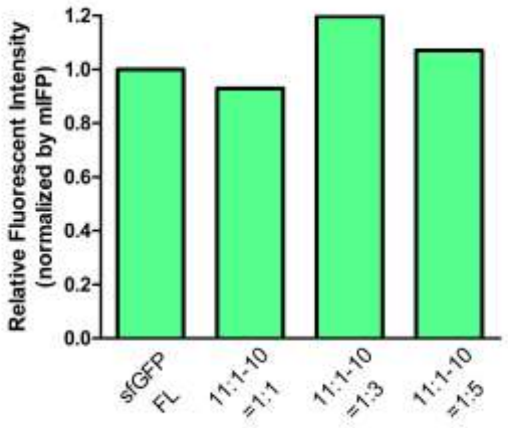

b

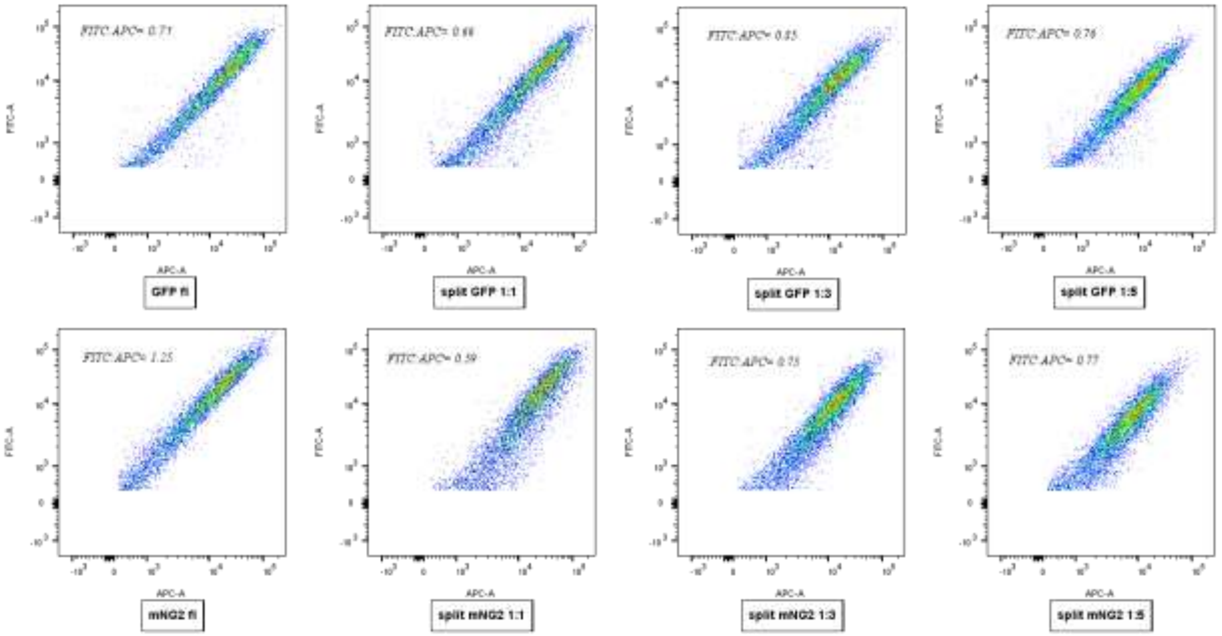

Supplementary Figure 1 (a) Whole cell fluorescence intensity of full length GFP and GFP_{11}-CLTA/$GFP_{1\text{-}10}$, measured by flow cytometry and normalized for expression level by mIFP fluorescence signal. Number of cells > 6000. **(b)** FACS raw data of bar graphs in Fig. 2e and Supplementary Fig. 1a. The X-axis is mIFP fluorescence intensity (ex=633 nm, em=710/50 nm) and the Y-axis is green fluorescence intensity (ex=488 nm, em=530/30 nm). The “FITC:APC” value is the population mean of green fluorescence normalized by mIFP fluorescence.

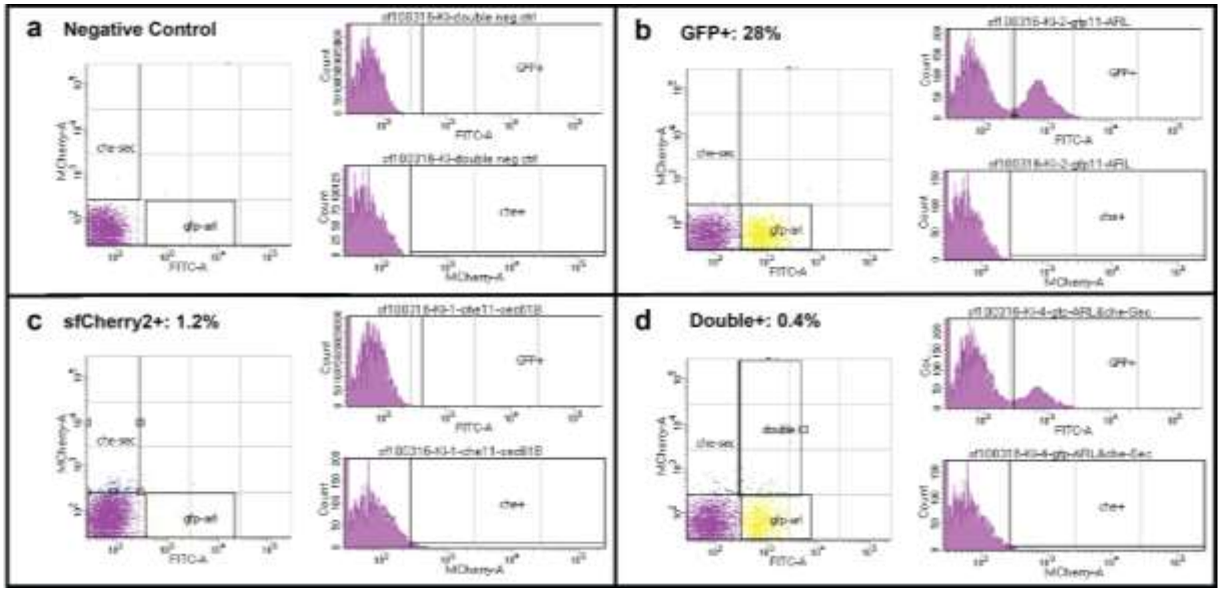

Supplementary Figure 2. FACS results of single knock-in or simultaneous double knock-in using GFP_{11} or/and $sfCherry2_{11}$. **(a)** Background fluorescence of HEK 293T cells stably expressing both $GFP_{1\text{-}10}$ and $sfCherry2_{1\text{-}10}$. **(b)** Single knock-in of GFP_{11} into ARL6IP1 with an efficiency of ~28% (GFP postitive). **(c)** Single knock-in of $sfCherry2_{11}$ into Sec61B with an efficiency of ~1.2% (sfCherry2 postitive). **(d)** Simultaneous double knock-in of GFP_{11} and $sfCherry2_{11}$ into ARL6IP1 and Sec61B respectively, with an efficiency of ~0.4% (double postitive).

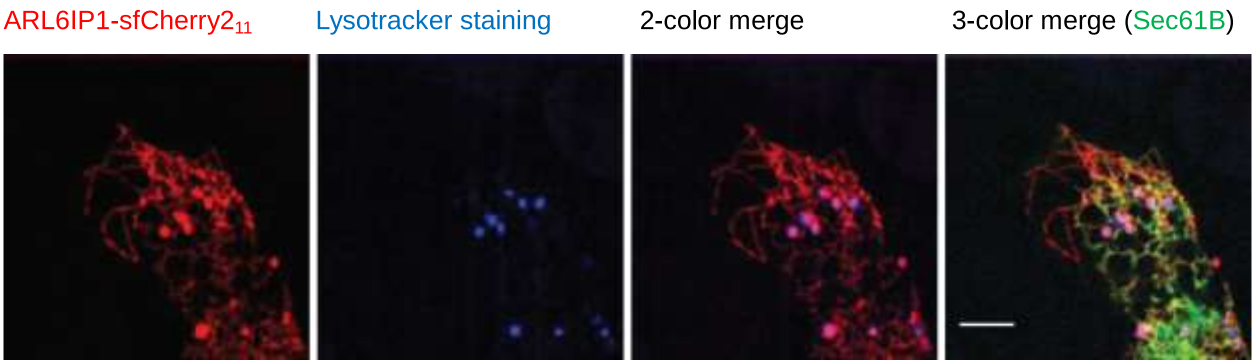

Supplementary Figure 3. Lysotracker live-cell staining visualize the colocalization of lysosomes and sfCherry2 puncta in the same ARL6IP1-sfCherry2$_{11}$/Sec61B-GFP$_{11}$ knock-in HEK 293T cell shown in Figures 6a and 6b. Scale bar: 5 μm.

Supplementary Table 1: Sequence of mNG2$_{1-10}$/mNG2$_{11}$, sfCherry2$_{1-10}$/sfCherry2$_{11}$, PAsfCherry2$_{1-10}$ and 32-residue spacer in the screening construct.

	DNA Sequence
**mNG2$_{1-10}$**	ATGGTGAGCAAGGGTGAGGAGGATAACATGGCCTCTCTCCCAGCGACTCATGAGTTACA<br>CATCTTTGGCTCCATCAACGGTGTGGACTTTGACATGGTGGGTCAGGGTACCGGCAATC<br>CAAATGATGGTTATGAGGAGTTAAACCTGAAGTCCACCAAGGGTGACCTCCAGTTCTCCC<br>CCTGGATTCTGGTCCCTCATATCGGGTATGGCTTCCATCAGTACCTGCCCTACCCTGACG<br>GGATGTCGCCTTTCCAGGCCGCCATGGTAGATGGCTCCGGATACCAAGTCCATCGCACA<br>ATGCAGTTTGAAGATGGTGCCTCCCTTACTGTTAACTACCGCTACACCTACGAGGGAAGC<br>CACATCAAAGGAGAGGCCCAGGTGATGGGGACTGGTTTCCCTGCTGACGGTCCTGTGA<br>TGACCAACACGCTGACCGCTGCGGACTGGTGCATGTCGAAGAAGACTTACCCCAACGA<br>CAAAACCATCATCAGTACCTTTAAGTGGAGTTACACCACTGTAAATGGCAAACGCTACCG<br>GAGCACTGCGCGGACCACCTACACCTTTGCCAAGCCAATGGCGGCTAACTATCTGAAGA<br>ACCAGCCGATGTACGTGTTCCGTAAGACGGAGCTCAAGCACTCCATG
**mNG2$_{11}$**	ACCGAGCTCAACTTCAAGGAGTGGCAAAAGGCCTTTACCGATATGATG
**sfCherry2$_{1-10}$**	ATGGAGGAGGACAACATGGCCATCATCAAGGAGTTCATGAGATTCAAGGTGCACATGGA<br>GGGCAGCGTGAACGGCCACGAGTTCGAGATCGAGGGCGAGGGCGAGGGCCACCCCTA<br>CGAGGGCACCCAGACCGCCAAGCTGAAGGTGACCAAGGGCGGCCCCCTGCCCTTCGC<br>CTGGGACATCCTGAGCCCCCAGTTCATGTACGGCAGCAAGGCCTACGTGAAGCACCCC<br>GCCGACATCCCCGACTACCTGAAGCTGAGCTTCCCCGAGGGCTTCACCTGGGAGAGAG<br>TGATGAACTTCGAGGACGGCGGCGTGGTGACCGTGACCCAGGACAGCAGCCTGCAGG<br>ACGGCCAGTTCATCTACAAGGTGAAGCTGCTGGGCATCAACTTCCCCAGCGACGGCCC<br>CGTGATGCAGAAGAAGACCATGGGCTGGGAGGCCAGCACCGAGAGAATGTACCCCGAG<br>GACGGCGCCCTGAAGGGCGAGATCAACCAGAGACTGAAGCTGAAGGACGGCGGCCAC<br>TACGACGCCGAGGTGAAGACCACCTACAAGGCCAAGAAGCCCGTGCAGCTGCCCGGC<br>GCCTACAACGTGGACATCAAGCTGGACATCACCAGCCACAACGAGGAC
**sfCherry2$_{11}$**	TACACCATCGTGGAGCAGTACGAGAGAGCCGAGGCCAGACACAGCACC
**PAsfCherry2$_{1-10}$**	ATGGAGGAGGATAACATGGCCATCATTAAGGAGTTCATGCGCTTCAAGGTGCACATGGAG<br>GGGTCCGTGAACGGCCACGTGTTCGAGATCGAGGGCGAGGGCGAGGGCCACCCCTAC<br>GAGGGCACCCAGACCGCCAAGCTGAAGGTGACCAAGGGTGGCCCCCTGCCCTTCACC<br>TGGGACATCCTGTCCCCTCAATTCATGTACGGCTCCAATGCCTACGTGAAGCACCCCGC<br>CGACATCCCCGACTACTTTAAGCTGTCCTTCCCCGAGGGCTTCACCTGGGAGCGCGTGA<br>TGAAATTCGAGGACGGCGGCGTGGTGACCGTGACCCAGGACTCCTCCCTGCAGGACG<br>GTCAGTTCATCTACAAGGTGAAGCTGCTGGGCATCAACTTCCCCTCCGACGGCCCCGTA<br>ATGCAGAAGAAGACCATGGGCTGGGAGGCCCTCACCGAGCGGATGTACCCCGAGGACG<br>GCGCCCTGAAGGGCGAGGTCAACCCGAGAGTGAAGCTGAAGGACGGCGGCCACTACG<br>ACGCTGAGGTCAAGACCACCTACAAGGCCAAGAAGCCCGTGCAGCTGCCCGGCGCCTA<br>CAACGTCGACCGCAAGTTGGACATCACCTCACACAACGAGGAC
**32-residue spacer**	GACGTTGGTGGTGGCGGATCAGAAGGAGGCGGTAGCGGGGGCCCTGGTTCGGGAGGG<br>GAAGGTTCTGCTGGGGGAGGGAGCGCTGGCGGGGGGTCT

Supplementary Table 2: sgRNA sequence

Target Gene	Target Term	Sequence of DNA oligo for sgRNA synthesis
SEC61B	N	TAATACGACTCACTATAGGCTTGTCTCCCTCTACAGCCGTTTAAGAGCTATGCTGGAA
LMNA	N	TAATACGACTCACTATAGGCCATGGAGACCCCGTCCCAGGTTTAAGAGCTATGCTGGAA
CLTA	N	TAATACGACTCACTATAGGGCCATGGCGGGCAACTGAAGTTTAAGAGCTATGCTGGAA
ARL6IP1	N	TAATACGACTCACTATAGGATCCCCGAGACGATGGCGGGTTTAAGAGCTATGCTGGAA
RAB11A	N	TAATACGACTCACTATAGGGTAGTCGTACTCGTCGTCGGTTTAAGAGCTATGCTGGAA
SPTLC1	C	TAATACGACTCACTATAGGGACTCTGCCTAGAGCAGGAGTTTAAGAGCTATGCTGGAA

Supplementary Table 3: Oligo-nucleotide donor DNA sequence

Target Gene-FP_{11} Tag	DNA sequence
LMNA-$mNG2_{11}$	TCCTTCGACCCGAGCCCCGCGCCCTTTCCGGGACCCCTGCCCCGC<br>GGGCAGCGCTGCCAACCTGCCGGCCATGACCGAGCTCAACTTCAAG<br>GAGTGGCAAAAGGCCTTTACCGATATGATGGGAGGTGGCATGGAGAC<br>CCCGTCCCAGCGGCGCGCCACCCGCAGCGGGGCGCAGGCCAGCT<br>CCACTCCGCTGTCGCCCA
CLTA-$mNG2_{11}$	CGGGCGTGGTGTCGGTGGGTCGGTTGGTTTTTGTCTCACCGTTGGT<br>GTCCGTGCCGTTCAGTTGCCCGCCATGACCGAGCTCAACTTCAAGG<br>AGTGGCAAAAGGCCTTTACCGATATGATGGGAGGTGGCATGGCTGAG<br>CTGGATCCGTTCGGCGCCCCTGCCGGCGCCCCTGGCGGTCCCGCG<br>CTGGGGAACGGAGTGG
RAB11A-$mNG2_{11}$	TGCAGCGACGCCCCCTGGTCCCACAGATACCACTGCTGCTCCCGCCCTTTC<br>GCTCCTCGGCCGCGCAATGACCGAGCTCAACTTCAAGGAGTGGCAAAAGGC<br>CTTTACCGATATGATGGGTTCTGGCGGCGGCACCCGCGACGACGAGTACGA<br>CTACCTCTTTAAAGGTGAGGCCATGGGCTCTCGCACTCTACACAGTC
SPTLC1-$mNG2_{11}$	GAACAAACAGAGGAAGAACTGGAGAGAGCTGCGTCCACCATCAAGGAGGTA<br>GCCCAGGCAGTTCTGCTCGGTGGCTCTGGCACCGAGCTCAACTTCAAGGAG<br>TGGCAAAAGGCCTTTACCGATATGATGTAGGCAGAGTCCCGGGACCATGGCC<br>TCCTGCCACACAACACGCAGAGAGGACTCAAGACTCCCGCTGGCCA
LMNA-$sfCherry2_{11}$	TCCTTCGACCCGAGCCCCGCGCCCTTTCCGGGACCCCTGCCCCGC<br>GGGCAGCGCTGCCAACCTGCCGGCCATGTACACCATCGTGGAGCAG<br>TACGAGAGAGCCGAGGCCAGACACAGCACCGGTGGCGGCGAGACC<br>CCGTCCCAGCGGCGCGCCACCCGCAGCGGGGCGCAGGCCAGCTC<br>CACTCCGCTGTCGCCCACCC
ARL6IP1-$sfCherry2_{11}$	GCGGGTTTCGGTTGGAGGACTCGTTGGGGAGGTGGCCTGCGCTTGT<br>AGAGACTGCATCCCCGAGACGATGTACACCATCGTGGAGCAGTACGA<br>GAGAGCCGAGGCCAGACACAGCACCGGTGGCGGCGCGGAGGGAG<br>ATAATCGCAGCACCAACCTGCTGGTGAGTCCTGGCTGCCTGTCCCCC<br>GGGAGCCGAGCGA
SEC61B- $sfCherry2_{11}$	GTGTCTAGGCCGGGGTTCTGGGGCAGGCCTGCCGCGCTCACCCGT<br>CTGTCTGCTTGTCTCCCTCTACAGTACACCATCGTGGAGCAGTACGA<br>GAGAGCCGAGGCCAGACACAGCACCGGTGGCGGCCCTGGTCCGAC<br>CCCCAGTGGCACTAACGTGGGATCCTCAGGGCGCTCTCCCAGCAAA<br>GCAGTGGCCGCCCGGGC
SEC61B-GFP_{11}	GTGTCTAGGCCGGGGTTCTGGGGCAGGCCTGCCGCGCTCACCCGT<br>CTGTCTGCTTGTCTCCCTCTACAGCGTGACCACATGGTCCTTCATGA<br>GTATGTAAATGCTGCTGGGATTACAGGTGGCGGCCCTGGTCCGACCC<br>CCAGTGGCACTAACGTGGGATCCTCAGGGCGCTCTCCCAGCAAAGC<br>AGTGGCCGCCCGGGC

sfCherry2$_{11}$-GFP$_{11}$-CLTA	AGCTGATAATACGACTCACTATAGGGCTCTGCAACACCGCCTAGACC<br>GACCGGATACACGGGTAGGGCTTCCGCTTTACCCGTCTCCCTCCTGG<br>CGCTTGTCCTCCTCTCCCAGTCGGCACCACAGCGGTGGCTGCCGGG<br>CGTGGTGTCGGTGGGTCGGTTGGTTTTTGTCTCACCGTTGGTGTCC<br>GTGCCGTTCAGTTGCCCGCCATGTACACCATCGTGGAGCAGTACGAG<br>AGAGCCGAGGCCAGACACAGCACCGGTGGCTCTGGAAGTTCAGGT<br>GGAGGCTCGCGTGACCACATGGTCCTTCATGAGTATGTAAATGCTGC<br>TGGGATTACAGGAGGCGGTATGGCTGAGCTGGATCCGTTCGGCGCC<br>CCTGCCGGCGCCCCTGGCGGTCCCGCGCTGGGGAACGGAGTGGC<br>CGGCGCCGGCGAAGAAGACCCGGCTGCGGCCTTCTTGGCGCAGCA<br>AGAGAGCGAGATTGCGGGCATCGAGAACGACGAGGCCT

mNG2$_{11}$ Sequence

sfCherry2$_{11}$ sequence

GFP$_{11}$ sequence

Linker sequence

Coding region sequence
